# Assessment of the Effectiveness of a Portable NIRS Instrument in Controlling the Mixer Wagon Tuning and Ration Management

**DOI:** 10.3390/ani11123566

**Published:** 2021-12-15

**Authors:** Lorenzo Serva, Luisa Magrin, Giorgio Marchesini, Igino Andrighetto

**Affiliations:** Department of Animal Medicine, Production and Health, University of Padua, Viale dell’Università 16, 35030 Padua, Italy; lorenzo.serva@unipd.it (L.S.); giorgio.marchesini@unipd.it (G.M.); igino.andrighetto@unipd.it (I.A.)

**Keywords:** total mixed ration, ration homogeneity, sorting activity, near-infrared, feed mixer wagon

## Abstract

**Simple Summary:**

The total mixed ration is widely adopted in herds feeding, and its success has paved the way for the use of the feed mixer wagon. The increase in milk production demands a higher feed efficiency that considers the chemical and physical quality of the ration, even in terms of fiber physical effectiveness, ration homogeneity, and cow feed sorting. These requirements presuppose a correct ration formulation and accurate preparation, even through an optimal mixer wagon setting. Here, we proposed an efficient, rapid, and easy method for ration preparation and quality control. A portable Near-InfraRed instrument was tested for primary chemical and physical evaluation of the ration, including the particle size and the physical effective fiber content. Moreover, the instrument allowed the calculation of two indexes to evaluate the ration homogeneity and cow sorting activity. Finally, these traits were compared with the feed mixer wagon setting and feed characteristics. As a result, we found that for the overload of the feed mixer wagon, the higher humidity and fiber contents of the ration caused a lower homogeneity, and the higher fiber content facilitated the cow sorting activity.

**Abstract:**

The adoption of the mixer wagon and total mixed ration aimed to decrease dysmetabolic diseases and improve feed efficiency in dairy cows. Differences between theoretical and eaten diets are imputable to errors in diet preparation or cow feed sorting. We proposed a method to measure the chemical composition and particle size distribution of the ration and determined its peNDF content through a portable Near Infra-Red spectrophotometer that allowed the calculation of two indexes: the homogeneity and the sorting indexes. In a cohort of 19 Italian Holstein breeding farms, we studied the correlation of these indexes with the mixer wagon settings. Determination coefficients in the validation (R_v_^2^) for dry matter, crude protein, aNDF, and starch were 0.91, 0.54, 0.86, and 0.67, respectively. The ration fractions (%, *w*/*w* of wet weight) retained by the 3.8 and 1.8 mm sieves, and the bottom showed R_v_^2^ of 0.46, 0.49, and 0.53, respectively. The homogeneity index regressed negatively with the mixer wagon load fullness (R^2^ = 0.374). The homogeneity-binary classification showed an odds ratio of 1.72 for dry matter and 0.39 for aNDF (*p* < 0.05). The sorting-binary classification showed an odds ratio of 2.54 for aNDF (*p* < 0.05). The studied farms showed low peNDF values (median = 17.9%).

## 1. Introduction

The total mixed ration (TMR) is not a novelty in dairy farming and has been reported in the scientific literature since the 1950s. The TMR was introduced to provide a consistent supply of nutrients to the rumen activity, optimize its functionality, and improve feed utilization. Differences between theoretical and actual feed intake have been reported as an unavoidable and detrimental effect in the daily production process that could affect milk yield, dry matter intake (DMI), and milk production efficiency [1]. In addition to the chemical composition of TMR, a consolidated requirement for an optimal TMR functionality is to reach the correct physical effective (pe) value of the NDF fraction, as initially defined by Mertens (1997) [2]. Traditionally, the pe value is estimated by using the Penn State Particle Separator (PSPS) [3,4].

In this context, ensuring the TMR homogeneity and particle size distribution assumes a preeminent role in the effectiveness of the herd feeding system [5]. The homogeneity of the ration in terms of particle size distribution is not always guaranteed across the year, and it is related to TMR composition [6]. Initially, the homogeneity of the distributed TMR was evaluated using a particle size separator and standard deviation (SD) as a test by sampling from 10 points at the feeding alley, attaining values of SD mostly lower than 20.0% [7]. Regarding the physical form of the ration, the particle size reduction of the forages in the TMR must be periodically monitored to ensure proper ruminal function in dairy cows [8]. The animal sorting activity has been reported as an unwanted circumstance [9,10], occurring mainly due to the ratio of concentrate to forage composition, the TMR processing time, the facility characteristics, and feed management [11,12,13].

The feed mixer wagons (MW) machines are used to prepare, transport, and deliver the TMR. Their manufacturing characteristics may influence the ration preparation accuracy, and the feed component separation is caused by the TMR-making procedure, including the mixing time and wear of the working parts responsible for cutting [14,15,16]. Additionally, the loading order level of filling the MW, the TMR preparation, the level of MW maintenance, the cutting-mixing time, and the moisture content of feedstuff are reported as limiting the correct TMR preparation [10]. In addition, in the TMR, the use of ingredients characterized by different densities causes feed segregation, where the heavier ingredients tend to sink and the lighter ones tend to float. The MW manufacturers often recommend the order and the time of adding ingredients, but the operators could accidentally outspread the mixing time, reducing the particle size of the TMR and favoring milk fat depression and other health problems [9]. The mixing time (MT) effect on the TMR dry matter (DM) homogeneity along the feeding alley has been previously reported [15]. In a previous study on beef cattle [13], a longer mixing time affected TMR homogeneity, decreased the particle size of the TMR and sorting activity of the bulls, and improved the bulls’ growth performances [13]. Resuming an undermixing may cause the TMR to be very inhomogeneous along the feed alley, while overmixing may cause an excessive particle size reduction [17]. Moreover, significant variability in the overall TMR [17] and in the feeding alley was already reported when analyzing three different points of the TMR distributed by MW for the main chemical constituent (DM, *p* < 0.01, and NDF *p* < 0.05), without regard to load fullness, mixing, or cutting time. This fact, coupled with cows’ behavioral habits to choose the same position at the feeding trough [18], highlights the differences in the TMR intake among the animals. Said variation within and between TMR batches requires an on-time analysis.

Definitively, the need to close the control loop by measuring the physical and chemical characteristics, including the particle size distribution of the TMR as it is delivered in the feed bunk, requires the monitoring of the uniformity among or within batches [10].

Near InfraRed Spectroscopy (NIRS) to evaluate the cow’s feed and ration, even in the form of the TMR, has been explored [19,20] for chemical [21] and physical traits [22,23]. The NIRS was confirmed to be effective in determining particle size distribution when coupled with chemometrics tools or even with Artificial Neural Networks (ANNs) [24], and it has long recognized the sensitivity of NIRS to particle size sample shape and distributions of the granular portion [25]. An attempt to determine a concise TMR homogeneity index and evaluate the animal’s sorting using NIRS has been successfully reported [22].

Under these circumstances, a comprehensive study on the effects of the MW and TMR chemical and physical characteristics on the ration homogeneity, cow sorting ability, and milk yield through a portable or MW-integrated NIRS instrument is still required. This study aimed to develop a method for evaluating the homogeneity and sorting attitude of the TMR through the calculation of two indexes, the Homogeneity index (Hi) and the Sorting index (Si). Furthermore, a portable NIRS instrument was calibrated for the chemical and physical traits of the TMR. Lastly, using the portable NIRS, we evaluated the relationships of MW and TMR characteristics with the Hi and Si in a cohort of 19 dairy farms.

## 2. Materials and Methods

### 2.1. NIRS Calibration and Internal Validation

A dataset of a cow’s TMR was built by collecting 311 samples from the Pianura Padana-Veneta country (North Italy) in the years from 2016 to 2018. Each untreated sample was scanned twice using a portable NIRS system (poliSPEC^NIR^, ITPhotonics, Fara Vicentina, Italy), and each scan lasted 10 s (with an integration time of about 10 ms). The average spectra were calculated for further use. The portable NIRS instrument was calibrated in a range of 902–1660 nm.

The reference methodologies adopted to calibrate the NIRS instrument were: #934.01 [26] for DM, 2001.11 [27] for CP; #996.11 [28] for starch; ANKOM Technology for aNDF, with amylase and sodium sulfite [29,30,31]; ANKOM Technology for nonsequential ADF [32,33].

Undried TMR samples were sieved twice, and the cumulative percentages were calculated for further use after data averaging by means of a Penn State Particle Separator (PSPS) modified through the addition of a sieve (PSPS-M). The sieving procedure was similar to that reported for the PSPS [3,4,17]. The PSPS-M was made of five sieves (from S1 to S5) and a bottom pan. Sieves 1 to 5 had squared holes with diagonals of 38.1, 19.1, 7.9, 3.8, and 1.8 mm. The geometric mean particle length (GMPL) was calculated as reported in Equation (1) [34]:

(1) $$\mathrm{GMPL}\;=e^{\left\{\left[\%S1\;\times \;\ln\left(48\right)\right]+\left[\%S2\;\times \;\ln\left(38,04\right)\right]+\left[\%S3\;\times \;\ln\;\left(17,32\right)\right]+\left[\%S4\;\times \;\;\ln\left(7,73\right)\right]+\left[\%S5\;\times \;\ln\left(3,69\right)\right]+\left[\%\mathrm{bottom}\;\times \;\ln\left(0,90\right)\right]\right\}}$$

The %S1 to %S5 and %bottom values reported in Equation (1) are the ratio (in percentage) of the original sample retained on each sieve (expressed as *w*/*w* of the wet sample). The cumulative percentages were corrected to obtain a sum of 100%.

Data from both chemical and physical analyses of TMRs were applied for NIRS calibration using SL Calibration Wizard (SensoLogic GmbH, Norderstedt, Germany). Spectra were submitted to pre-treatments as the Standard Normal Variate (SNV), the Detrending [35], the Smoothing, and the 1st derivative [36]. Per calibrated trait, the dataset was randomly split into calibration (c = 55%) and validation (v = 45%) sets by the software’s internal function. Thus, the v-set accounted for different selected samples among traits. The calibration was carried out by a Partial Least Square (PLS) algorithm on the c-set after two cross-validation steps and outliers’ detection. Outliers’ detection was based on the use of the Mahalanobis distance (outliers marked as samples with M-Distance value > 3.0) and the Cook’s D statistic (D outliers > 3) as proposed by the software and reported in Equation (2):

(2) $$D\;=\frac{T^{2}\times H}{\left(k+1\right)\times \left(1-H\right)}$$

where *T* = studentized residuals; *H* = leverage; *k* = number of model variables. The maximum number of factors was set to 10, and the final choice was made by minimizing the Root-Mean-Square Error (RMSE).

The calibrations were evaluated by the coefficient of determination in cross-validation (R^2^_cv_), the RMSE in cross-validation (RMSE_cv_), the Index of Random Variation (IRV), the Index of Systematic Variation (ISV), the F-value (the ratio of variance explained by the model over residual variance), and the Skew (the deviation in the slope of the regression line in a plot of actual versus predicted values from the ideal value of 1). Finally, all calibrations were evaluated for the validation set, calculating the coefficient of determination (R^2^_v)_, the Root-Mean-Square Error (RMSE_v_), and the GH (the global Mahalanobis) in the validation set.

### 2.2. Farm, TMR, and Mixer Wagon Data Collection

An observational open cohort study of 19 dairy farms was conducted over two years (from 2019 to 2020). Each farm was visited with a median of 2 (1–3, first to third quartile range, 57 visits in total), within a period of 50, 163, and 393 days (medians) for 2, 3, or 4 consecutive visits. The dairy farms were located in the Pianura Padano-Veneta Region, north of Italy (Figure S1). Farms, breeding Friesian-Holstein cows, were recruited from a list of eligible farms provided by a feeding company. Their participation was voluntary. At each visit, trained researchers supported by farmers completed a survey and, using a portable poliSPEC^NIR^, ran the analysis of the ration provided to the lactating cows’ group. The survey was intended to collect data on the MW characteristics, TMR formulation, herd composition, and milk yield. The latter was intended as the average milk yield on the week of the farm visit and calculated at the farm level. The dry matter intake (DMI) was calculated at the farm level, only for the pluriparous cows’ group, as the total ration amount (at the net of declared residuals) was divided by the number of cows and corrected for the DM (%) content of the ration. Sixteen points located at regular intervals on the feeding alleys were analyzed per visit using the NIRS, immediately after TMR distribution and after 2 h at the same points. The physical effective aNDF (peNDF) [2] was calculated by the NIRS predicted values of the aNDF (% of DM) of the TMR, and further corrected by its cumulative fraction with a length ≥ 4.00 mm [17,37]. The MW load fullness was evaluated as the ratio (%) of the loaded TMR volume to the declared MW volume of the tank. The loaded TMR volume was calculated by the TMR weight (ton) × its specific weight (ton/m^3^). The latter was previously estimated by weighing a TMR sample contained in a 2 L volume.

The NIRS predictions for physical and chemical TMR traits were used to calculate the TMR Hi and Si. The Hi (ranging from 0 to 100%, 100% = perfect homogeneity) was calculated as the weighted sum of the standard deviation to mean ratios of TMR composition recorded along the feeding alley at 16 points after outliers’ removal and compared with the maximal acceptable ratios (arbitrary values). Outliers were considered as values exceeding the mean ± 2 standard deviations. The valuable traits for Hi calculation and corresponding weights and acceptable ratios (values are reported in brackets) were S4 (8, 3%), S5 (22, 3%), bottom (22, 5%), GMPL (20, 10%), CP (10, 3%), aNDF (9, 3%), and starch (9, 3%). The Si was evaluated at the same 16 points applied for Hi, but fresh-distributed TMR analyses were compared with those collected from the same 16 points after 2 h from feed distribution. A *t*-test was performed by comparing fresh TMR vs. 2 h later analyses within the same traits used for the Hi calculation. The calculated *p*-values were arbitrarily associated with a grid of discrete values (Table 1), ranging from 0 to 1, where zero means no sorting and one means total sorting. The latter values were weighted as for Hi and finally added together. The latter value was divided by 100 to obtain a parameter ranging from 0 (minimal sorting) to 1 (maximal sorting).

In Figure S2 of the Supplementary Materials, a schematic flowchart of the whole trial is drawn.

### 2.3. Statistical Analysis

Data collected from repeated visits at the same farm were averaged before statistical analysis, avoiding excessive recorded data fluctuation. Continuous variables were evaluated for normality assumption by the Shapiro–Wilk test, visual inspection of the frequency distribution, and the Q–Q plot (quantile–quantile plot). Nonnormally distributed data were reported as the median and first and third quartiles, whereas normally distributed data were reported as the mean and standard deviation (SD). A two-sided, two-sample, Student’s *t*-test was calculated for the S4, S4 + S5 (C5), and S4 + S5 + bottom (Cb) values (% of the wet weight of TMR) to compare actual vs. NIRS predicted values. The median (approximate values) was used as a threshold for Hi and Si binary classification as inhomogeneous (Ibhi, Hi ≤ 79%) or homogeneous (Hbhi), and as negligible selection (NSbsi, Si ≤ 0.30) or evident selection (ESbsi, Si > 0.30). The median milk yield at the farm level was used to group the farms in low (FMY-low) or high (FMY-high) yield. Logistic and linear models assessed the outcomes (Hi; Si) for predictors, and Adjusted R^2^ and Residual Standard Error (RSE) evaluated the goodness of the regression. The Variance Inflation Factor (VIF) measured the multicollinearity of the predictor of the equations. Receiver Operating Characteristic (ROC) analysis was performed using the reference indexes as the gold standard to calculate the model’s accuracy. The ROC curve is the plot of sensitivity and 1-specificity, and the area under that curve (AUC) represents the accuracy of the test showing its effectiveness. The correlation between variables was calculated as Pearson (for quantitative variables) or Spearman (for qualitative variables) correlation coefficients. Only the correlations with r ≥ 0.6 were reported, with few exceptions.

Principal Component Analysis (PCA) was performed over the mean MW and physical and chemical traits of the TMR for the 19 selected farms of the cohort. The first ten principal components were calculated after scaling the variables, and the first two were used to plot the loadings of the original variables to the new latent variables.

All statistics were performed using R version 4.0.2 (22 June 2020).

## 3. Results

### 3.1. NIRS Calibration for Chemical and Physical Traits of TMR

**Figure 1:**
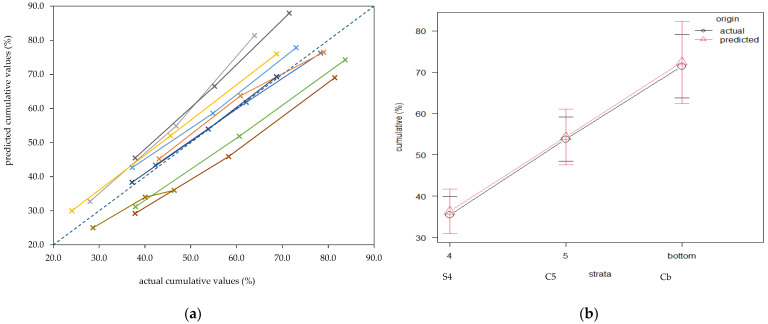
(**a**) Cumulative (%) NIRS predicted vs. actual values for the lower strata (S4, C5, and Cb) of the modified PSPS-M, for the ten samples chosen within the validation (v) set. The dotted line represents the perfect fitting. (**b**) The S4, C5, and Cb cumulative values of total mixed ratio wet weights plotted for NIRS predicted vs. actual values for the ten samples chosen within the v set. The vertical lines represent the 95% confidence limits for the evaluated trait.

The RMSE for the two repeated sieves of the PSPS-M were 6.63, 3.52, 4.26, 4.22, 1.89, and 3.42% for S1, S2, S3, S4, S5, and bottom, respectively. The mean values were 2.30, 7.79, 20.0, 34.2, 17.9, and 17.8% for S1, S2, S3, S4, S5, and bottom, respectively, while the average GMPL was 6.73 mm. The calibration results for the chemical and physical traits are reported in Table 2. For ten validating samples, the S4, C5, and Cb cumulative fractions were compared to the NIRS predicted values and are plotted in Figure 1a. The ten samples were selected among those in the validation set, all having available data for S4, S5, and bottom. The cumulative values (S4, C5, and Cb) resulted as normally distributed for actual and NIRS predicted data. Actual vs. NIRS predicted values resulted in 35.4 vs. 36.3 (*p* = 0.79), 53.8 vs. 54.3 (*p* = 0.95), and 71.5 vs. 72.4 (*p* = 0.86) for S4, C5, and Cb, respectively. The regression for predicted vs. NIRS actual values of S4, C5, and Cb cumulative values of TMR wet weights (%) is shown in Figure 1b, and described in Equation (3), reporting an adjusted R^2^ = 0.81 (*p* < 0.001, RSE = 7.80).

(3) NIRS predicted =2.80+0.96×actual 

### 3.2. Farm, TMR, and Feed Mixer Wagon Data Results

The MW characteristics, TMR formulation, herd composition, and milk yield data are reported in Table 3.

The Hi resulted as negatively correlated with the loading (kg/s) or the auger speed (RPM) for the single feed (loading alfalfa r =−0.31, auger speed alfalfa r = −0.58, loading speed concentrate r = −0.72, auger speed concentrate r = −0.59, loading speed for nonmaize silages r = −0.67, auger speed for nonmaize silages r = −0.52), and slightly with the GMPL (r = −0.34). The Si resulted as negatively correlated with the auger speed (RPM) for maize silage (r = −0.77), with the starch content of the TMR (r = −0.72), with the loading (kg/s), and with the auger (RPM) speed for concentrate (r = −0.68 and r = −0.66, respectively), while it correlated positively with the aNDF content of the TMR (r = 0.61), and slightly with GMPL (r = 0.33). The DM content of the TMR was negatively correlated with the GMPL (r = −0.71), MW fullness (r = −0.54), and feed intake (r = −0.87), but positively with the DMI (r = 0.21). The peNDF was negatively correlated with the DM content of the TMR (r = −0.47), the loading (kg/s), the auger (RPM) speed for alfalfa (r = −0.29 and −0.88, respectively), the auger speed (RPM) for concentrate and nonmaize silage (r = −0.88, and −0.47), the loading time (r = −0.71), and total operating time (r = −0.56), while it positively correlated with the GMPL (r = 0.41) and the feed intake (r = 0.79).

**Figure 2:**
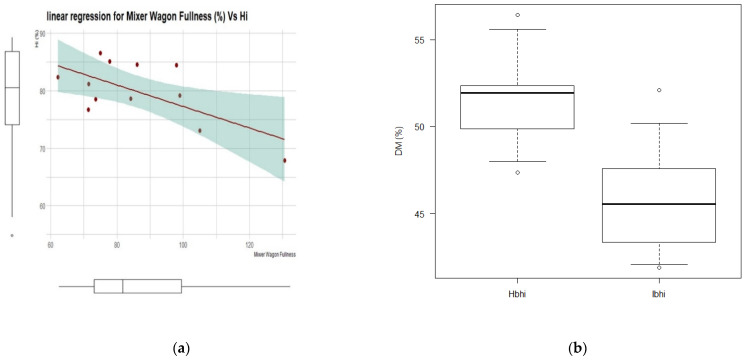

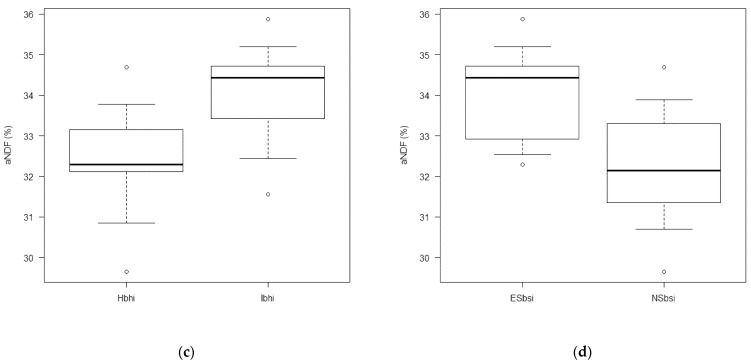
(**a**) Linear regression for Hi vs. Mixer Wagon Fullness (%); the grey area represents the 95% confidence interval, the full red line represents the least squares regression line; the abscissa and ordinate of the Whiskers box-plot with median, first-third quartile, and 5–95 percentile for represented data; (**b**) the Whiskers box-plot for the ration Dry Matter means compared for Homogeneity index (Hi) binary classes (Hbhi, Hi > 79%; Ibhi, Hi ≤ 79%); (**c**) the Whiskers box-plot for the ration aNDF means compared for Hi binary classes (Hbhi, Hi > 79%; Ibhi, Hi ≤ 79%); (**d**) the Whiskers box-plot for the ration aNDF means compared for Si binary classes (NSbsi, Si ≤ 0.30; ESbsi, Si > 0.30).

Data collected from the farm, TMR, and mixer wagon were evaluated to estimate the Hi and Si values through simple linear regression (continuous variable) or logistic (binary variable), and the Hi and Si classification according to Ibhi/Hbhi and NSbsi/ESbsi classes, respectively. Only regressions showing *p* < 0.05 are further reported. With regard to the Hi continuous value, the linear regression for mixer wagon load fullness showed (Hi = 96.0 − 0.19 × MW fullness) an adjusted R^2^ = 0.374 (*p* = 0.02), RSE = 4.33 (Figure 2a). Regarding Hi binary classification, the logistic regression for DM showed odds ratio = 1.72 (*p* = 0.03) and AUC = 0.89, and the DM means were 52.7 and 45.9 (*p* = 0.003) for Hbhi and Ibhi, respectively (Figure 2b). The logistic regression for aNDF showed odds ratio = 0.39 (*p* = 0.06) and AUC = 0.79, and the aNDF means were 32.4 and 34.0 (*p* = 0.038) for Hbhi and Ibhi, respectively (Figure 2c).

Considering the Si binary classification, the logistic regression for aNDF showed odds ratio = 2.57 (*p* = 0.06) and AUC = 0.82, and the aNDF means were 32.2 and 34.0 (*p* = 0.035) for Hbhi and Ibhi, respectively (Figure 2d).

The regression between Hi and Si showed a negative trend (Si = 0.52 − 0.0035 Hi, *p* = 0.305) with a very poor Adjusted R-squared = 0.008.

A multiparametric linear regression was tested to estimate the milk yield considering the Hi and Si of the ration showing an Adjusted R-squared = 0.226 (*p* = 0.195), RSE = 2.336, and VIF = 1.004 for farms with FMY-low, and Adjusted R-squared = 0.202 (*p* = 0.245), RSE = 1.318, and VIF = 1.566 for farms with FMY-high (Equations (4) and (5)).

(4) Milk yield (FMY − low)=22.4+0.01×Hi−11.2×Si 

(5) Milk yield (FMY−high)=47.6−0.12×Hi−2.74×Si

(6) Milk yield =32.8+7.37× number of MW augers +5.74× MW type [towed]– 0.50× MW volume 

**Figure 3:**
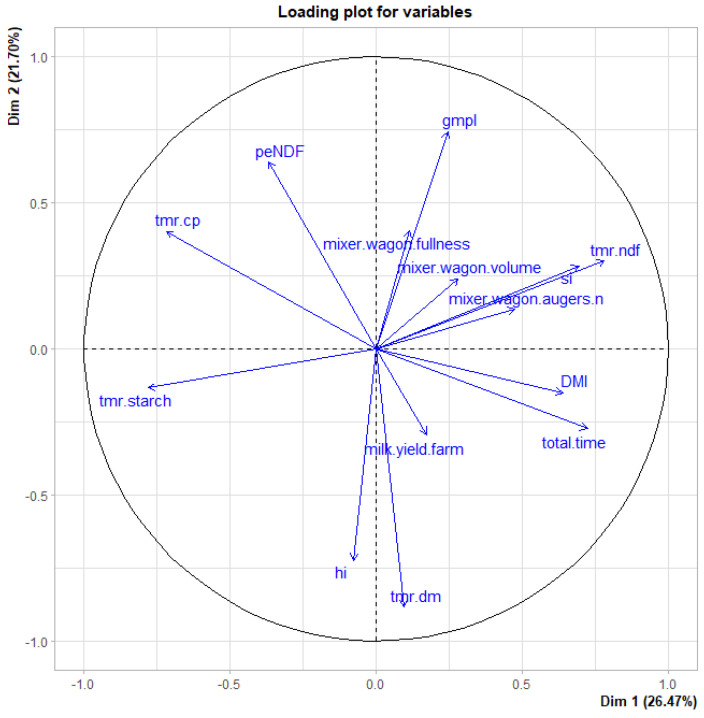
The first two PCs (Dim 1 and 2) plot for the dataset of the 19 farms of the evaluated cohort. The first ten latent variables were calculated upon the MW setting (total time operating, volume, augers number, fullness), TMR physical (peNDF (% of the DM), Geometric Mean Particle Size (GMPL, mm)) and chemical traits (DM, CP, aNDF, starch content (% of the DM)), Dry Matter Intake (DMI, kg of DM) and milk yield at farm level (kg/day at farm level). Dim 1 and 2, the first two PC, counted for 26.47 and 21.70% of the explained variance, respectively.

The PCA allowed a visual evaluation of the relations between predictors (Figure 3). The first 5 PCs counted for 26.5, 21.7, 14.8, 9.87, and 8.11% of the explained variance. TMR features included the Hi. The Si showed the higher PC-1 and -2 values. The coordinates, cos^2^, and contributions of the single variable and supplementary groups are reported in Table S1. The Hi appeared close to the DM and partially to the milk yield, with a negative value for PC2. Along the PC2, the Hi was opposed to GMPL, peNDF, and a trimmer distance with MW fullness. The Si matched with aNDF and some MW settings (volume and number of augers), while it was opposed to starch.

A multiparametric linear regression was tested to estimate the milk yield by the use of the MW characteristics and times (loading time, mixing time, total operating time, number of MW augers, MW fullness MW type, MW volume), while the remaining traits were excluded to ensure VIF < 10.0. At the AIC backward/forward criterion, only the number of MW augers, MW type, and MW volume were selected to obtain a final multiparametric linear regression, showing an Adjusted R-squared = 0.155 (*p* = 0.271), RSE = 3.43, and AIC = 30.1 (Equation (6)).

## 4. Discussion

The effectiveness in predicting essential nutrients, such as CP, NDF, starch, NFC, and fat of the TMR, was already proven with valuable R^2^_cv_ (>0.85) in dried ground samples [20]. Here, the use of the NIRS for the evaluation of the fresh ungrounded TMR evidenced valuable results for the main chemical traits. The better R^2^_v_ was obtained for DM, aNDF, ADF, and starch. The DM calibration showed similar results compared with a previous work, where the NIRS was applied to the MW predicted grass silage or maize silage DM content [38]. Better results were achieved for DM and aNDF calibrations when compared with the analysis of maize silage without any sample preparation and the use of a petri dish as a sample holder [39]. Poor results were obtained for CP. The prediction of CP shows lower performance than previous work, where the CP showed R^2^_v_ = 0.71 [39]. The results are generally lower than those reported using an equivalent-spectral-range portable NIRS applied to dried-ground forages. At the same time, they are better than those obtained through a narrow-spectral-range portable instrument [40]. Moreover, our results are generally similar to those in cross-validation obtained for fresh hand-harvested natural fresh pastures [41] by the use of Fourier-Transform Near-InfraRed Spectroscopy (FT-NIRS) with a spectral range of 4000–9999 cm^−1^ (1100–2500 nm), except for CP and ADF (reported R^2^_cv_ = 0.88 and 0.82, respectively). Further, a lab-scale NIRS instrument (covering a spectral range of 1100–2500 nm) was reported to be calibrated for fresh grass with a lower R^2^_v_ for DM or higher for CP (0.85, and 0.83, respectively) [42].

Following previously accepted classification thresholds [43,44], our R^2^_v_ was classified as excellent for DM, good for aNDF and ADF, quite good for starch, and quite useful for CP, while the remaining calibrated traits were lowest. Our findings confirmed the crucial role of the sample preparation for calibration accuracy. Nevertheless, several repeated scans might reduce the sampling errors, especially for inhomogeneous products.

Cross-sensitivity is the ability of NIRS to measure two quantities, i.e., the particle size and the chemical composition. In conventional NIRS application, the first is an unwanted signal that would be minimized, grounding the sample [25]. Here, we wanted to exploit particle size analysis by appreciating the effect of the so-called light scattering [25]. The results reported in Figure 1a,b showed valuable performances and R^2^ for NIRS, predicting cumulative fractions (S4, C5, and Cb) compared with actual values. Moreover, it is noticeable that the RMSE_cv_ are only 1.29, 1.56, 1.85, and 1.31 greater than the RMSE evaluated in PSPS-M reference measures for S3, S4, S5, and bottom, respectively. Even though the reported RMSE by using undried and ungrounded samples in NIRS analysis was higher than those from bench NIRS instruments predicting dried-ground samples, the intrinsic ability of the portable NIRS might be considered to increase the number of analytical repetitions of analysis for fresh ungrounded samples. Increasing the number of repeated scans will reduce the absolute error; i.e., using the *t*-student table, rising from a single to 16 repeated NIRS analyses as for the Hi calculation, will reduce the 95% confidence interval of the error from 12.7 to 2.12 × standard error of the mean. These findings confirm the ability and usefulness of the calculated NIRS calibration for a rapid evaluation of ration preparation within the use of the MW. This fact assumes relevance in feed analysis where significant variations in silage or hay DM content and the ration intake are reported [10].

Concerning the recorded TMR fractions of the 311 calibration samples, the S1 + S2 cumulative mean value was slightly higher than the 2–8% suggested value for the upper 19 mm PSPS sieve. Moreover, the S3 mean value was lower than the 30 to 50% suggested for the 8 mm middle PSPS sieve, the S4 mean value was higher than the 10 to 20% suggested for the 4 mm PSPS sieve, and finally, the recorded average bottom showed a lower content compared with the maximum percentage values of 30–40% suggested by PSPS [17]. Moreover, in our findings, the mean value for the TMR classified as physically effective (>4 mm) was 64%, according to the PSPS suggestion of 60 to 70% [17]. Regarding the GMPL, we found an average value lower than 9.19–11.55 mm reported by Grant [45] but similar to those reported in a previous study [37].

Regarding the cohort’s herd, the TMR [46,47,48] and the MW characteristics [18] are consistent with a typical medium-high level milk yield farm in northern Italy. The mixer wagon fullness ranged from 73.1 to 98.2% in the first to third quartile, consistent with data reported in the literature, while the recorded mixing time was considerably lower [15,18]. The recorded peNDF values were lower than those previously reported by Serva et al. (2021) [37] (aNDF = 32.9 and peNDF = 26.2% of the DM with the GMPL = 6.13 mm) or by Grant et al., 2020 [45], reporting the GMPL ranging from 9.19 to 11.55 (aNDF = 34.6 and peNDF = 20.6% of the DM). The peNDF values in our findings are partially explained by the lower GMPL than those reported in the cited literature. The suggested threshold of peNDF > 21% [2] was not achieved in most farms studied in the cohort. The average milk yield was higher than those reported in studies carried out under similar conditions [49] or similar at comparable days in milk [47]. The lack of TMR preparation, the low peNDF, and the low Hi values are probably due to the uncorrected overload of the MW. The Hi highlighted good values for many farms, but the first quartile showed Hi lower than 74.0. These findings are opposed to those found in the literature where authors measured the TMR homogeneity, evaluated as the SD of the proportion of feed particles on the separator screens, which was good (SD < 20%) for eight MW regardless of the different designs of the working elements [7]. The Hi was mainly related to the MW fullness, confirming the ideal reference range of 80–100 [15,18], with a severe undesirable Hi with fullness > 100% (Figure 2a). Overall, the animal sorting resulted as moderate, but data confirmed the cow capability to sort, probably due to their ability to select against particles retained above sieves with holes >18 mm and to simultaneously choose particles passing through the 1.65 mm screen, regardless of the effect of the alfalfa content, as reported in the literature [12,50]. Results from Figure 2a–c confirmed that TMR levels of DM [50] and aNDF [15,18] were related to Hi (positively for DM and negatively for aNDF), as well as to the Si (positively for aNDF). Surprising the DM content was a risk factor for the Hbhi, probably because farmers tended to overmix and overcut the TMR with higher DM, as proven by the negative correlation between DM and the GMPL, as well as the negative correlation between DM and peNDF (primarily due to the pef coefficient of the peNDF value, as the DM was uncorrelated with the aNDF). The MW setting for TMR preparation correlated with the final physical quality of the ration.

Here, we did not find a relation between the mixing time and the fiber length; however, from Figure 2 and the correlation values, the recorded total operating time of MW was opposed to peNDF, which means that in the case of shorter operating time, a higher fiber fraction is retained by the upper sieves. These findings confirm results reported in the literature where the authors found a trend for the fiber ratio in the PSPS upper sieve when MT ≤ 17 min, while a lower amount of feed was found in the middle sieve and the bottom compared to MT > 17 min [15], and a significant variation both in chemical and physical composition (*p* < 0.05) was observed over days. The same authors noted that when MT ≤ 17 min, the DM was lower at the beginning-middle of the feeding alley than the final part. There was no apparent effect of the operating time on the Hi values, but this lack of results can also be due to the large number of variables influencing the outcomes. It is noticeable that cutting is prolonged along with the entire MW operations and can be influenced by several factors. Finally, the threshold of 17 min values was recorded at the first quartile of the total operating time but only at the third quartile of the mixing time. The auger and the loading speeds were negatively correlated with Hi, Si, and peNDF, probably due to shorter particle size. Moreover, lower values of GMPL led to a higher Hi and lower Si. These findings confirmed the role of the MW setting in the correct physical proprieties of the TMR, which resulted as insufficient compared with the suggested minimum threshold of peNDF (21%) [2], but higher than the 14.1% values reported in a previous Italian study [51], where a lower PSPS retaining rate (0.39%) was found at the 19 mm sieve. The lower GMPL confirmed this trend compared with values reported in the literature [6,17,45]. According to findings reported in the literature, the Si was negatively correlated with the milk yield [50,52]. The Si was positively correlated with starch and negatively with aNDF, probably due to a most prolonged preparation time of a more fibrous TMR. The PCA approach confirmed these findings, suggesting the relevant role of the TMR in explaining the dataset variability and the interaction with the MW settings.

Even though the reported results from Equations (4) and (5) were not significant but tendential, the Hi and Si had different influences on milk yield and seemed more relevant for FMY-low farms, probably due to prevalent errors in the ration management. Equation (6) demonstrated that the MW setting scarcely affected the milk yield. Due to the limited number of farms and repetitions recorded in this study, these findings cannot evaluate the factors influencing the milk yield but might underline the importance of monitoring the effectiveness of the ration preparation.

Despite the suitable results obtained from the NIRS calibration, particularly regarding the PSPS-M validation, some limitations in the study must be underlined. The number of farms in the cohort must be improved to represent the Italian’s farming characteristics, and more repetitions must be performed within the farm to covariate seasonal effects and to be able to assess productivity traits better. Moreover, correlations are frequently close to 0.6, and regressions to milk yield are challenging to interpret, requiring deeper investigation and confirmation. The loading sequence of feed could play a role in the TMR homogeneity and should be recorded to improve the knowledge in terms of ration management. Finally, the digestibility of the nutrients should be evaluated according to Hi and MW-ration management. However, this newly proposed method, including new indexes (Hi and Si) and the NIRS, a fast and nondestructive technique, will allow the researchers to efficiently collect a large amount of data and better describe the relationships among MW characteristics and indexes.

Moreover, the proposed method will help the manufacturing producers better understand the MW potentials and the farmers tune their MW correctly in relation to the TMR characteristics. The NIRS, Hi, and Si can be installed in the MW as optional tools, perfectly fitting the idea of precision livestock farming (PLF), especially in the coming new digital era where much data will be managed to control the dairy production process better. Nevertheless, the indexes (Hi and Si) themselves should not be intended as the final point of the research. Moreover, the farmers could not understand the reasons and effects of such indexes. More in-depth studies should be done, such as evaluating the relations between the indexes and the digestibility of the rations.

## 5. Conclusions

Errors in feeding preparation should be quickly identified and corrected. The variability in feeds, primarily silages and forages, and the uncertainty in the preparation procedures require a daily evaluation of the TMR composition. The MW should be correctly tuned to warrant a high ratio of TMR homogeneity. The homogeneity and sorting values for the TMR are evaluated typically using a particle separator, while the chemical composition is usually assessed with a lab-scale NIRS instrument; both are uncomfortable and inappropriate tools to be used as continuous evaluation systems at the farm level. Our findings demonstrated that a portable NIRS is accurate enough to be used at the farm level as a daily and rapid screening tool for fresh ungrounded ration to predict the leading chemical and physical characteristics, including the Hi and Si. The Hi and Si were proposed to evaluate some MW characteristics tuning, and their values were satisfactory in the studied cohort. The Hi was related to the aNDF and DM contents, while the Si to the aNDF. We could observe that farmers using a high-DM-content TMR tended to overcut the ration, and most of the studied farms had a low-peNDF-content TMR. Further research should be performed to improve the dairy farms cohort’s variability and deeply evaluate the relations among the studied variables. Moreover, the Hi and Si should be examined according to the ration digestibility and the milk yield.

## Figures and Tables

**Table 1:** Arbitrary assignment for *p* calculated in the *t*-test for the 16 points evaluated in Sorting index (Si) calculation, comparing fresh vs. 2 h later analyses.

*p* ^1^	Arbitrary Assignment Value
0.00 < *p* ≤ 0.01	1.0
0.01 < *p* ≤ 0.05	0.5
0.05 < *p* ≤ 0.10	0.4
0.10 < *p* ≤ 0.20	0.2
0.20 < *p* ≤ 0.50	0.1
*p* > 0.50	0.0

^1^ Observed *p*.

**Table 2:** Calibration results for chemical and physical traits in the NIRS cross-validation (cv) and validation (v) datasets.

Model	Ncal	PC	OR	F-val	ISV	IRV	RMSE_cv_	R^2^_cv_	Nval	S	RMSE_v_	R^2^_v_	GH
Chemical Traits													
Dry Matter	110	5	0	366	1562	1828	1.38	0.93	53	0.89	1.85	0.91	0.98
Crude protein	113	5	1	15.8	−306	531	0.74	0.27	49	0.88	0.73	0.54	1.58
aNDF	107	5	2	98.2	−192	1444	1.47	0.78	51	1.14	1.44	0.86	1.06
ADF	115	5	2	76.1	128	988	1.01	0.72	43	1.04	1.02	0.81	1.27
Starch	115	5	0	34.4	1575	1369	1.36	0.50	47	0.96	1.16	0.67	0.93
Sieves													
S1 (%)	194	5	24	26.9	1140	1433	1.69	0.31	93	6.42	17.6	0.53	1.48
S2 (%)	206	3	12	21.9	269	535	3.75	0.20	93	1.38	6.56	0.17	1.15
S3 (%)	218	4	0	25.0	341	1588	5.27	0.24	93	1.06	5.51	0.33	1.02
S4 (%)	217	5	1	56.1	−4448	3854	6.65	0.50	93	0.97	6.61	0.46	0.91
S5 (%)	216	5	2	58.0	1340	3077	3.41	0.52	93	0.89	3.50	0.49	1.03
Bottom (%)	214	5	4	58.1	4437	4029	4.63	0.52	93	1.05	4.49	0.68	1.62
GMPL (mm)	205	5	13	47.4	−541	2027	2.00	0.48	93	1.66	4.04	0.53	1.27

Ncal = number of samples in calibration; PC = factors used in calibration; OR = number of outliers removed; F-val = the ratio of variance explained by the model over residual variance; ISV = index of systematic variation; IRV = index of random variation; RMSE_cv_ = root-mean-square error in cross validation; R^2^_cv_ = coefficient of determination in cross validation; Nval = number of samples in validation; S = skew; RMSE_v_ = root-mean-square error in validation; R^2^_v_ = coefficient of determination in validation; GH = global Mahalanobis distances for samples in validation; S1 to S5 are the ratios (in percentage) of the original sample retained on each sieve, as *w*/*w* of the wet sample; GMPL = the geometric mean particle length.

**Table 3:** The feed Mixer Wagon (MW) characteristics, Total Mixed Ratio (TMR) formulation, herd composition, and milk yield for the 19 selected farms of the open cohort underwent the survey.

	Mean	SD	IQR	Min	I-Q	II-Q	III-Q	IV-Q	N	NA
Herd pluriparous										
- cows (n)	254	234	133	79.3	128	203	262	999	14	5
- dry matter intake (kg of the DM)	23.2	1.59	1.20	19.7	22.7	23.3	23.9	25.8	17	2
- total intake (kg of the as-is of TMR)	47.2	7.51	2.00	38.0	44.8	46.2	46.8	65.1	9	10
- average milking days	175	17.6	22.0	140	164	174	186	215	17	2
- milk yield (kg/day)	33.8	3.78	5.48	26.8	31.0	35.0	36.5	39.0	18	1
Total Mixed Ration										
- homogeneity index (%)	76.7	9.76	10.24	55.9	74.0	78.9	84.3	86.7	18	1
- sorting index (pure number)	0.25	0.13	0.16	0.04	0.17	0.27	0.33	0.50	17	2
- geometric mean particle length (mm)	6.14	0.87	1.13	4.90	5.56	6.04	6.69	7.69	18	1
- dry matter (% of the DM)	49.2	4.35	6.04	41.9	46.1	49.4	52.1	56.4	16	3
- crude protein (% of the DM)	15.2	0.62	0.68	14.0	14.8	15.1	15.5	16.4	16	3
- starch (% of the DM)	26.0	1.18	1.93	24.6	25.0	25.6	26.9	28.8	16	3
- aNDF (% of the DM)	33.1	1.61	2.35	29.6	32.2	33.1	34.5	35.9	16	3
- peNDF (% of the DM)	18.1	4.73	7.28	10.0	14.4	17.9	21.7	25.6	15	4
Auger speed on loading (RPM)										
- alfalfa	22.3	8.16	11.50	10.0	17.3	24.5	28.8	30.0	6	13
- concentrate	20.6	8.79	15.0	10.0	12.5	24.0	27.5	30.0	7	12
- maize silage	20.0	6.96	9.50	10.0	15.0	20.0	24.5	30.0	7	12
- other silages	18.7	6.98	6.50	10.0	15.0	17.5	21.5	30.0	6	13
- overall average	22.7	4.47	5.00	15.0	20.0	23.0	25.0	30.0	9	10
Augers number				1.00		2.00		2.00	19	0
Loader speed (RPM)										
- alfalfa	300	64.7	76.3	200	265	313	341	375	6	13
- concentrate	212	17.0	12.0	200	206	212	218	224	2	17
- maize silage	304	45.2	76.3	250	265	313	341	350	6	13
- other silages	319	81.3	121	200	266	333	388	400	6	13
Loading speed (kg/s)										
- alfalfa	2.99	0.53	0.26	2.50	2.68	2.94	2.94	4.00	6	13
- concentrate	15.6	18.3	12.1	2.80	4.46	8.75	16.59	50.90	6	13
- maize silage	15.3	2.23	2.38	12.1	13.8	15.9	16.2	18.9	7	12
- other silages	9.23	3.58	4.54	4.00	7.50	8.89	12.0	13.5	6	13
Total loading time (s)	1697	587	630	993	1200	1615	1830	3300	17	2
Operating total time (s)	2192	529	760	1200	1800	2048	2560	3300	17	2
Mixing time (s)	561	331	250	180	400	535	613	1600	15	4
Mixer wagon fullness (%)	86.2	19.1	25.1	62.2	73.1	81.0	98.2	131	12	7
Mixer wagon volume (m^3^)	25.2	4.83	9.00	16.3	20.0	26.0	29.0	33.0	19	0
Mixer wagon type										0
- self-propelled									15	
- towed									4	

SD = standard deviation; IQR = interquartile range (first to third); min = minimum; I-Q to IV-Q = first to fourth quartile; N = number of farms counted for; NA = missing values.

## Data Availability

Data available upon request to the corresponding author.

## Supplementary Materials

The following are available online at https://www.mdpi.com/article/10.3390/ani11123566/s1, Figure S1: Farms location in the Pianura Padano Veneta country, Figure S2: A schematic flowchart of the entire trial procedures, Table S1: Coordinates, contributes, and cos2, for individual variables used to perform the PCA and supplementary categories for the 19 farms studied in the cohort.
