# Supplementary material for: Assessment of the Effectiveness of a Portable NIRS Instrument in Controlling the Mixer Wagon Tuning and Ration Management
Source: Animals (Basel). 2021 Dec 15;11(12):3566. doi: 10.3390/ani11123566 (PMC8698189; doi:10.3390/ani11123566)
Supplement: Supplementary file 1 [file animals-11-03566-s001.zip › animals-1458454-supplementary.pdf]

animals

MDPI

Supplementary materials

Figure S1: Farms location in the Pianura Padano Veneta country. Dati cartografici ©2021 Google,GeoBasis-DE/BKG (©2009)

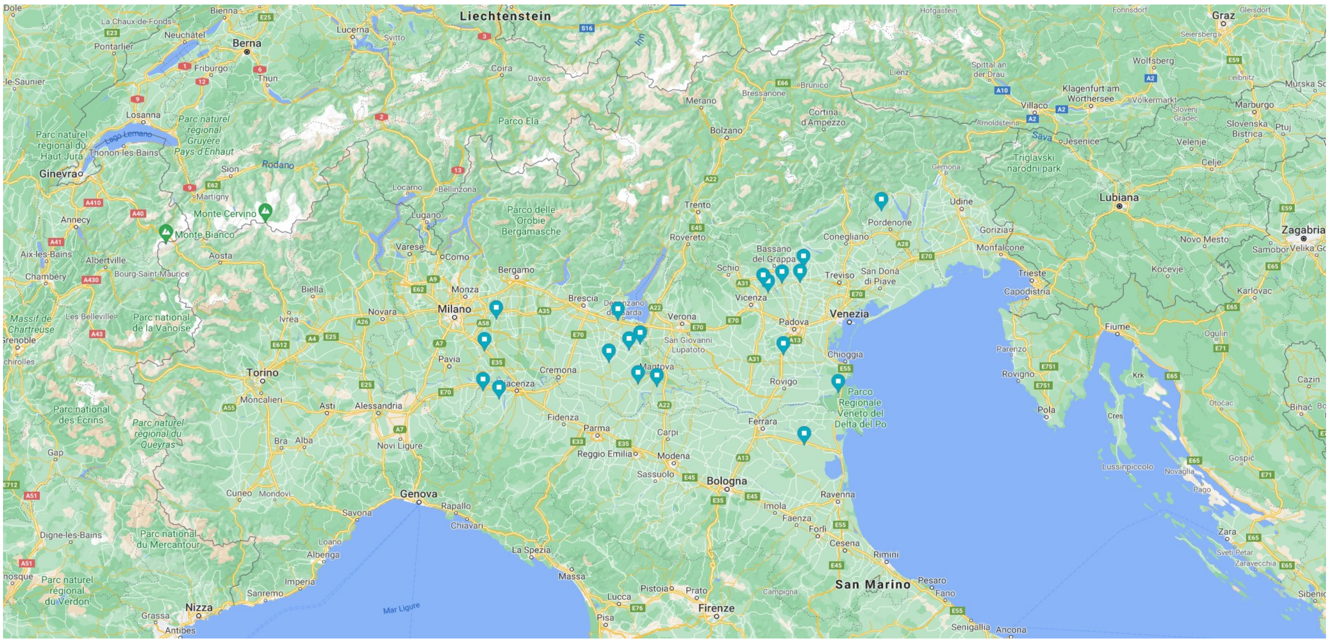

Figure S2: A schematic flowchart of the entire trials procedures

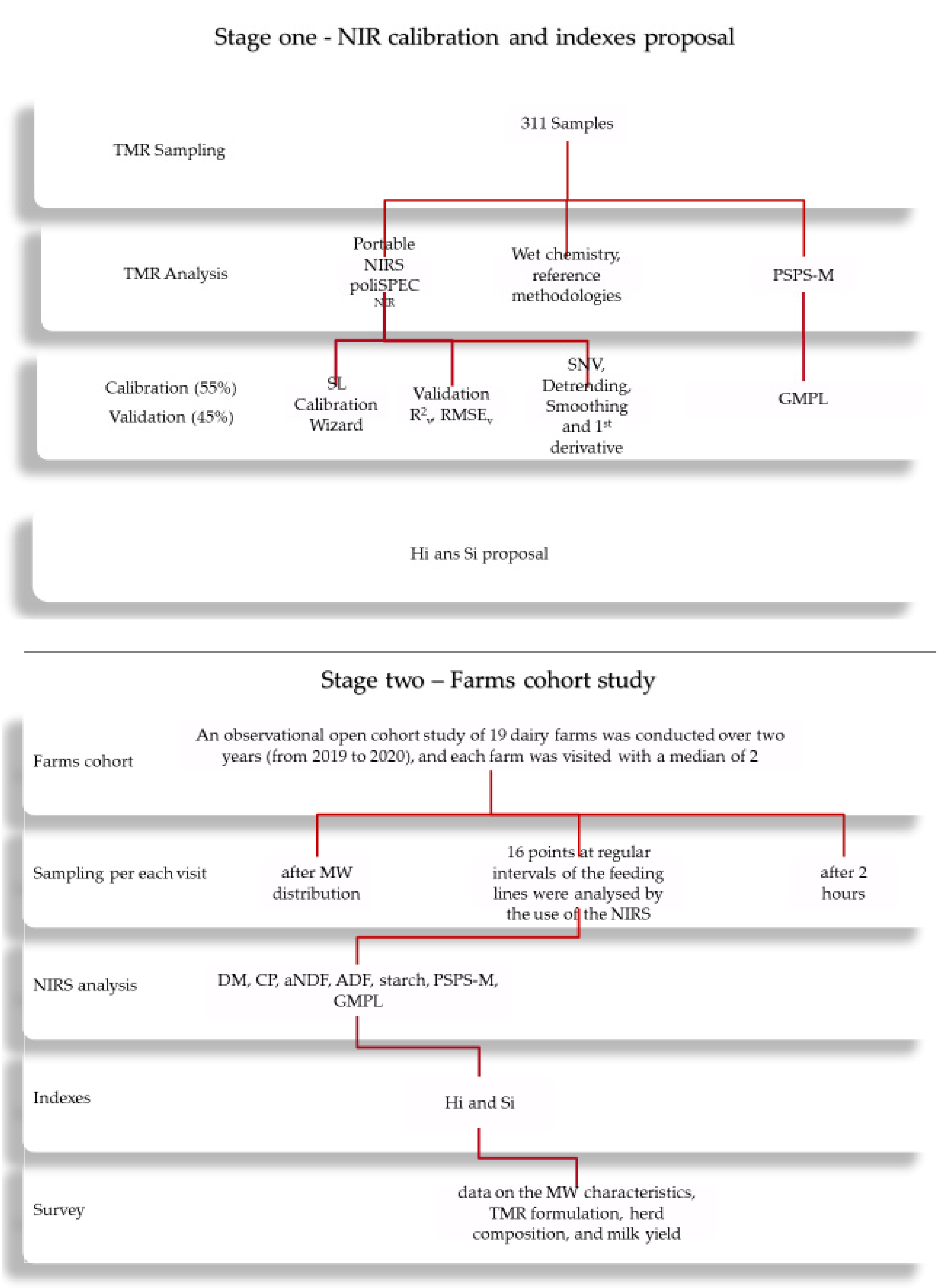

Dry Matter (DM), Crude Protein (CP), Geometric mean particle length (GMPL), Homogeneity index (Hi), Sorting index (Si), total mixed ration (TMR), Coefficient of determination in validation (R^2_v), Root mean square error in validation ($RMSE_v$), Standard normal variate (SNV)

Table S1: Coordinates, contributes, and cos^2, for individual variables used to perform the PCA and supplementary categories for the 19 farms studied in the cohort.

Variables															
	Dim.1	ctr	cos2	Dim.2	ctr	cos2	Dim.3	ctr	cos2	Dim.4	ctr	cos2	Dim.5	ctr	cos2
MW.augers.n	0.48	6.08	0.23	0.14	0.61	0.02	0.54	13.9	0.29	-0.60	25.8	0.36	0.11	1.13	0.01
MW.volume (m^3)	0.28	2.11	0.08	0.24	1.90	0.06	0.68	22.5	0.47	-0.51	19.1	0.26	0.05	0.25	0.00
MW.total.time (sec)	0.72	14.1	0.52	-0.27	2.46	0.08	0.02	0.02	0.00	0.13	1.13	0.02	-0.36	11.4	0.13
Hi	-0.08	0.17	0.01	-0.72	17.2	0.52	0.27	3.57	0.07	0.11	0.88	0.01	0.39	13.2	0.15
Si	0.69	13.0	0.48	0.28	2.65	0.08	-0.17	1.30	0.03	0.37	9.98	0.14	0.19	3.10	0.04
GMPL (mm)	0.25	1.62	0.06	0.74	18.2	0.55	-0.10	0.52	0.01	0.02	0.02	0.00	0.32	9.04	0.10
milk.yield.farm (kg/day)	0.17	0.81	0.03	-0.29	2.85	0.09	0.67	21.4	0.45	0.45	14.6	0.20	0.00	0.00	0.00
mixer.wagon.fullness (%)	0.12	0.35	0.01	0.41	5.40	0.16	0.39	7.37	0.15	0.24	4.05	0.06	-0.65	36.9	0.42
DMI (Kg of the DM/day)	0.64	11.0	0.41	-0.15	0.74	0.02	0.49	11.7	0.24	0.33	7.78	0.11	0.06	0.27	0.00
DM (%)	0.10	0.25	0.01	-0.88	25.6	0.78	-0.01	0.01	0.00	0.01	0.00	0.00	0.19	3.18	0.04
aNDF (% of the DM)	0.78	16.5	0.61	0.30	3.00	0.09	-0.36	6.18	0.13	-0.07	0.35	0.01	-0.02	0.04	0.00
CP (% of the DM)	-0.72	13.9	0.52	0.40	5.31	0.16	0.40	7.52	0.16	0.02	0.02	0.00	0.15	2.05	0.02
Starch (% of the DM)	-0.78	16.4	0.61	-0.13	0.59	0.02	0.15	1.06	0.02	-0.01	0.01	0.00	-0.35	10.9	0.12
peNDF (% of the DM)	-0.37	3.65	0.14	0.64	13.53	0.41	0.25	3.02	0.06	0.47	16.2	0.22	0.31	8.48	0.10

Supplementary categories																
	Dim.1	cos2	v.test	Dim.2	cos2	v.test	Dim.3	cos2	v.test	Dim.4	cos2	v.test	Dim.5	cos2	v.test	Dist
MW self-propelled	-0.28	0.41	-1.20	0.03	0.00	0.14	-0.06	0.02	-0.35	-0.15	0.12	-1.06	0.16	0.13	1.20	0.44
MW towed	1.05	0.41	1.20	-0.11	0.00	-0.14	0.23	0.02	0.35	0.57	0.12	1.06	-0.58	0.13	-1.20	1.63
Hbhi	-0.36	0.08	-0.74	-1.04	0.67	-2.40	0.26	0.04	0.73	0.01	0.00	0.02	0.43	0.11	1.61	1.27
Ibhi	0.34	0.07	0.70	1.03	0.66	2.38	-0.30	0.05	-0.82	0.04	0.00	0.12	-0.44	0.12	-1.64	1.27
ESbsi	1.19	0.62	2.24	0.61	0.16	1.27	-0.38	0.06	-0.96	0.33	0.05	1.01	0.36	0.06	1.23	1.51
NSbsi	-1.31	0.76	-2.73	-0.60	0.16	-1.39	0.10	0.01	0.29	-0.27	0.03	-0.94	0.04	0.00	0.16	1.50
FMY-low (kg/day)	-0.27	0.05	-0.56	0.04	0.00	0.08	-0.95	0.63	-2.65	-0.62	0.27	-2.12	-0.16	0.02	-0.59	1.19
FMY-High (kg/day)	0.25	0.04	0.52	-0.04	0.00	-0.10	0.92	0.60	2.56	0.66	0.31	2.26	0.15	0.02	0.56	1.18

DM, dry matter of the TMR; CP, crude protein of the TMR; Hi, homogeneity index, Si, sorting index; GMPL, geometric mean of the particle size (mm); Ibhi (Inhomogeneous Hi ≤ 79%); Hbhi (homogeneous); NSbsi (negligible selection Si ≤ 0.30); ESbsi (evident selection, Si > 0.30); FMY-low: Farm milk yield < 35 kg/day; FMY-high: Farm milk yield ≥ 35 kg/day; MW, mixer wagon set.
